# From anonymity to “open doors”: IRB responses to tensions with researchers

**DOI:** 10.1186/1756-0500-5-347

**Published:** 2012-07-03

**Authors:** Robert Klitzman

**Affiliations:** 1 Department of Psychiatry, Columbia University, 1051 Riverside Drive #15, New York, NY, USA

**Keywords:** IRBs, Research ethics, Responsible conduct of research, Ethics education, Professionalism, Medical education, Organizational ethics, Compliance, Communication

## Abstract

**Background:**

Tensions between IRBs and researchers in the US and elsewhere have increased, and may affect whether, how, and to what degree researchers comply with ethical guidelines. Yet whether, how, when, and why IRBs respond to these conflicts have received little systematic attention.

**Findings:**

I contacted 60 US IRBs (every fourth one in the list of the top 240 institutions by NIH funding), and interviewed leaders from 34 (response rate = 55%) and an additional 12 members and administrators. IRBs often try to respond to tensions with researchers and improve relationships in several ways, but range widely in how, when, and to what degree (e.g., in formal and informal structure, content, and tone of interactions). IRBs varied from open and accessible to more distant and anonymous, and in the amount and type of “PR work” and outreach they do. Many boards seek to improve the quantity, quality, and helpfulness of communication with PIs, but differ in how. IRBs range in meetings from open to closed, and may have clinics and newsletters. Memos can vary in helpfulness and tone (e.g., using “charm”). IRBs range considerably, too, in the degrees to which they seek to educate PIs, showing them the underlying ethical principles. But these efforts take time and resources, and IRBs thus vary in degrees of responses to PI complaints.

**Conclusions:**

This study, the first to explore the mechanisms through which IRBs respond to tensions and interactions with PIs, suggests that these committees seek to respond to conflicts with PIs in varying ways – both formal and informal, involving both the form and content of communications. This study has important implications for future practice, research, and policy, suggesting needs for increased attention to not only *what* IRBs communicate to PIs, but *how* (i.e., the tone and the nature of interactions). IRBs can potentially improve relationships with PIs in several ways: using more “open doors” rather than anonymity, engaging in outreach (e.g., through clinics), enhancing the tone as well as content of interactions, educating PIs about the underlying ethics, and helping PIs as much and proactively as possible. Increased awareness of these issues can help IRBs and researchers in the US and elsewhere.

## Background

Conflicts between institutional review boards (IRBs) – or research ethics committees (RECs), as they are called in many countries – and researchers have mounted over recent decades, yet little systematic attention has been given to critical questions of whether IRBs acknowledge and respond to these tensions, and if so, how, when, and why. In the US and elsewhere, IRBs have been increasingly faulted for several reasons, including extension of their review into the social sciences [1], and discrepancies in reviews of the same protocol in multi-site studies, delaying research, and impeding comparison of data [2]. Critics contend that IRBs have become overly bureaucratic, focused on the ethics of documentation [3], and may be “dyfunctional” [4]. These problems frustrate researchers, generating tensions. In other countries as well, IRBs have been criticized – e.g., as inefficient [5].

As a result, for two decades, debates have arisen over whether the *status quo* should be altered, and if so, how. Critics have call for increased centralization [6,7], though in the US, this alternative has been instituted only in limited ways. In July 2011, The US Office of Management and Budget released an Advance Notice of Proposed Rule Making (ANPRM) recommending changes to 45-CFR-46 (the so-called “Common Rule”), the federal regulations governing IRBs [8,9]. The ANPRM addresses several issues, reflecting in part researchers’ complaints about IRBs. Specifically, the document seeks to increase central review; reduce variations between IRBs that can impede research; allow some minimal risk research to be “excused” from IRB review; and address challenges raised by biobanking. But whether any of these possible changes in formal structural elements of IRB reviews will be made, and if so, which, to what degree, in what form, and when, is unclear. Critical questions arise, too, of whether other changes may be needed or beneficial as well in improving the current system.

Importantly, though PIs’ complaints about IRBs have been described [1,2,6,10,11], little, if any, attention has been given to whether IRBs respond to these critiques, and if so, how. Yet researchers’ beliefs that IRBs are unfair may dissuade these researchers from fully adhering to research ethics guidelines [12]. Logistical aspects of IRBs have been examined (e.g., sociodemographics of members, and time required for IRB approval [2,13]), but whether IRBs decide to address strains with researchers, and if so, how, and to what degree, have not been systematically examined.

As part of a qualitative, in-depth interview study of IRB chairs, focused on understanding their views, attitudes, and roles regarding research integrity (RI), broadly defined [14], many issues arose – e.g., concerning differences in how IRBs made decisions and interacted with PIs, and viewed and approached conflicts of interest [15], central IRBs [16], research in the developing world [17], and variations between IRBs [18]. Yet, other separate issues arose concerning how IRBs addressed interactions and conflicts with PIs, and tried to improve these relationships. Since qualitative research allows for further probing of themes that arise, these interviews then explored these mechanisms in greater detail. Crucial questions emerged of how IRBs responded to PIs, and what approaches facilitated and/or impeded their relationships. This paper thus analyzes and explores these realms.

## Methods

As described elsewhere [14], in-depth telephone interviews of approximately 1 to 2 h each were conducted with 46 chairs, directors, administrators, and members. The leadership of 60 IRBs (every fourth one in the list of the top 240 institutions by NIH funding) was contacted and IRB leaders from 34 of these institutions were interviewed, yielding a response rate of 55%. In certain cases, both a chair/director as well as an administrator from an institution were included (e.g., if the former thought that the latter could better provide detail about certain areas). Thus, in all, 39 chairs/directors and administrators from these 34 institutions were interviewed. To understand the impact of varying social and institutional milieus in these domains, institutions ranged in location, size, and public/private status. Every other interviewee was also asked to disseminate information about the study to their IRB members, in order to also recruit 1 member from each IRB. Seven other members were thus included, as well.

As summarized in Table 1, the 46 interviewees included 28 chairs/co-chairs; 10 administrators (including 1 director of a compliance office); and 7 members. In all, 58.7% were male, and 93.5% were White. Interviewees were distributed across geographic regions, and institutions by ranking in NIH funding. This study was approved by the Columbia University Institutional Review Board. All interviewees gave informed consent.

**Table 1:** Characteristics of the sample

	**Total**	**% (N = 46)**
**Type of IRB Staff**		
Chairs/Co-Chairs	28	60.87%
Directors	1	2.17%
Administrators	10	21.74%
Members	7	15.22%
**Gender**		
Male	27	58.70%
Female	19	41.30%
**Institution Rank**		
1–50	13	28.26%
51–100	13	28.26%
101–150	7	15.22%
151–200	1	2.17%
201–250	12	26.09%
**State *vs.* Private**		
State	19	41.30%
Private	27	58.70%
**Region**		
Northeast	21	45.65%
Midwest	6	13.04%
West	13	28.26%
South	6	13.04%
**Total # of Institutions Represented**	**34**	

Appendix A presents relevant portions of the semi-structured interview guide, which sought to elucidate interviewees’ aspects of their decisions, lives, and social situations by trying to grasp their own experiences and language, not by imposing theoretical structures [19]. The methods draw on elements from grounded theory [20].

After completion of all of the interviews, a trained research assistant (RA) and the principal investigator (PI) conducted additional analyses in two phases. In the first phase, each interview was read, and “core” codes or categories were assigned to blocks of text (e.g., instances of IRB interactions and tensions with PIs). Together, these independently-developed coding schemes were then reconciled. A coding manual was produced, listing and defining the codes. Any areas of disagreement were explored until consensus was reached. Issues that did not fit in the original coding manual were discussed, and modifications were made when necessary.

In the second phase of the study, the two coders independently content-analyzed the interviews, examining the main subcategories, and ranges of variation in each of the core categories. They reconciled sub-themes into a single set of “secondary” themes and an elaborated set of core categories. Sub-themes included, for example, specific types of interactions with researchers (e.g., use of memos, face-to-face meetings), PI reactions (e.g., PIs’ complaints about the IRB to institutional leadership), and IRB efforts to reduce conflicts with PIs (e.g., changing the tone of memos sent).

Codes and sub-codes were then used in analysis of all of the interviews, with the two coders analyzing all interviews.

## Results

**Figure 1:**
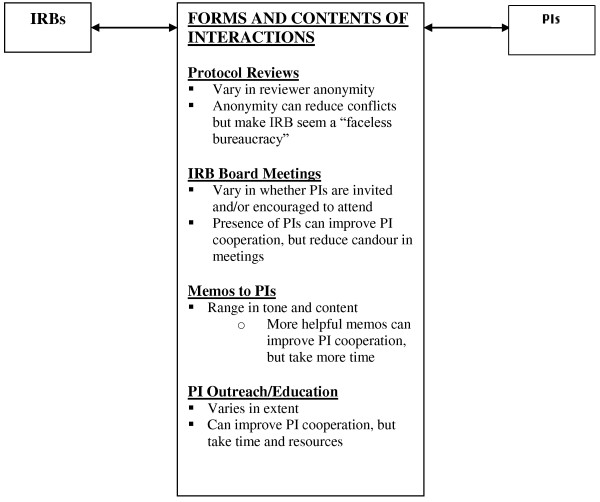
Responses of IRBs to tensions with PIs.

As summarized in Figure 1, and described more fully below, IRBs face several choices regarding both the structure and content of interactions with PIs. These committees often try to respond to tensions with PIs, and improve relationships in various ways, but range in how, when, and to what degree. IRBs varied in the formal and informal social structures, and the content and tone of interactions with PIs, and confronted several challenges.

### From anonymity to open doors

In general, IRBs differed in the types, amounts, and effectiveness of their efforts, and ranged across a spectrum from remaining distant and anonymous, to being open and accessible. IRB members often said they knew they were seen as “obstructionistic” by PIs, but these interviewees varied in how much they were troubled by, and responded to, these perceptions. Chairs generally said that they were supportive of PIs, but they differed widely in how, and to what degree they demonstrated that stance. IRBs often adopted approaches to try to reduce tensions, but these methods then had both strengths and limitations that IRBs confronted.

Some IRB leaders suggested that they were highly attentive to PI views, and that they tried to be “open” and helpful, while others expressed these concerns much less. For example, some IRBs tried to be very open in both the form and tone of the interaction.

"Our approach here is: “Please call.” Not: “We’re out to get you.” With a lot of IRBs, the relationship and rapport they have with the faculty causes the problem. PIs don’t want to deal with them, as opposed to picking up the phone and trying to get some information. That’s just a general philosophy, tone, and culture. **IRB26**"

IRBs may thus differ in both their implicit and explicit attitudes and practices.

#### IRB anonymity

In contrast, other interviewees suggested that their committees remained more distant from PIs. Much of IRB work occurs behind closed doors, and chairs may try to shield IRB members from researchers’ criticism by keeping these members anonymous. Such anonymity can result from several causes. For instance, a reviewer may have a relatively low position in the institutional hierarchy, and not want to offend superiors on whom his or her future may depend.

"One scientist was new to the IRB, and was appalled at a colleagues’ poorly thought-out protocol, but unwilling to go to him outside of the meeting, and make suggestions. The PI probably wouldn’t have listened, so we just kind of limped along with it. **IRB26**"

This anonymity can thus create tensions, protecting the reviewer, but potentially delaying or hampering streamlining of a review. To avoid friction with PIs, many IRBs have reviewers of specific protocols remain anonymous.

"At our institution, the cancer researchers know who the cancer reviewers are – from whom the feedback comes. However, friction from that confrontation is held to a minimum. We give members *an option* of whether to reveal themselves or not when communicating to PIs prior to committee meetings. We respect reviewers’ desire for anonymity and confidentiality. If members have questions for a PI, they can go through IRB staff instead. Otherwise, our IRB membership lists are provided to PIs along with our correspondence, making the names of the reviewers known. **IRB9**"

Regarding openness to PIs, IRBs may develop their own group processes and culture that can be difficult to change. A chair may try to alter an IRB by adding members who, he or she feels, will optimally perform their tasks. The chair can also dismiss other members. The interviewee above wants members to “reach out” to PIs, and he has:

"…removed any member from the IRB who wants to remain permanently anonymous, because we require that board members make an effort to reach out to our PIs. That’s just pure old marketing, and good will with our researchers. That’s how we change the perception here of the IRB. **IRB9**"

Some IRBs may thus see themselves as having to actively “market” their services (i.e., to engender support from researchers).

When he revamped the IRB, to improve relationships with researchers, this director asked a dozen members to leave.

"When we reorganized, we removed 12 members, who had been on the IRB for many years, because most of them had no desire to communicate with investigators prior to meetings. One of the prerequisites to then becoming a member was willingness to reach out and contact PIs prior to committee meetings. At times, they kept confidentiality when it was a personal colleague, or friend, or someone they work with closely. That’s OK. **IRB9**"

IRB members can thus vary in their interest and willingness to communicate with PIs – i.e., the degrees to which they would “reach out.”

Questions arise of whether IRB decision-making processes should be more transparent to not only a particular PI, but more broadly. IRBs keep minutes private, along with all correspondence and decisions (except to the PI involved). Yet at times, certain interviewees felt that heightened transparency could potentially also improve perceptions of IRBs among PIs.

"Minutes are not now publicly available, but *should* be. I don’t see why not. I guess researchers may feel it’s embarrassing to have your stuff rejected. **IRB22**"

Some interviewees felt that redaction of details at any institution may also be hard, but it is not possible.

#### Outreach and public relations (PR) work

IRBs varied in the amount and type of “PR work” and outreach they do with PIs, with some boards working hard to convey the message that “we’re not the enemy.” As one chair said, “Our educational sessions have helped. Because the IRB members are *human beings*.” (IRB4) IRBs may thus try to shape their image and alter the notion that they are a faceless bureaucracy, rather than consisting of fellow individuals.

At times, interviewees felt that PIs blamed IRBs for trying to make protocols conform to federal regulations. An IRB may try to establish “good PR” to help reduce this problem by making itself less impersonal and anonymous.

"Our IRB has done years of public relations work all over campus saying, “We’re not the enemy. We’re not here to hurt you. We’re here to help you, to talk with you. Tell us what problems you’re having. How can we assist?” That hasn’t always been the case here.** IRB39**"

IRBs and institutions may also thus try to change their approaches over time.

Some IRBs go further, asking PIs how the IRB can improve its interactions and relationships. In these efforts, IRBs can be very strategic – being proactive, and targeting wary researchers.

"We have identified departments that are particularly hard to work with, and said, “Can we come talk to you?…Let us know how we can work with you.” We go make presentations to faculty meetings, project coordinators, or individual faculty members: “How can we make this better for you?” **IRB39**"

Certain IRBs, recognizing that they are viewed warily by PIs, explicitly try hard to alter these negative perceptions, to give the message, “We’re not *devils*. We’re doing a good job” (IRB27) – seeking to reverse this metaphor of embodying evil (i.e., “devils”).

Chairs differ in these efforts, which can take time and effort. New chairs may adopt methods that are new to an institution. One started calling new PIs.

"I had some tools that this campus hadn’t seen before. I just picked up the phone and said, “Can I come and show you what this is all about? Because I know you’re not going to know.” And they were fine with it. I tell them it’s going to make it a whole lot easier. **IRB21**"

He also invites PIs to educate the IRB, which he thinks works well.

"If we have new methodologies, protocols – where the committee is frankly just ignorant – they start out gray. We usually invite that investigator to come in – if they have an illustration or tools to help us understand. Then the committee can get educated. From a PR standpoint, that’s helped, because investigators feel we’re willing to be taught, and reach out. **IRB21**"

#### Open door policies

Many chairs seek to improve relationships with PIs through the structure and amount of other kinds of interactions as well, striving to maintain “*open door” policies*, making themselves directly and highly accessible to PIs via email, phone, or cell phone. These types of physical and logistical *structures* of personal interaction may shape psychological and social attitudes and vice versa. But the nature, extent, and rationales involved varied.

Here, too, chairs may play critical roles, and prod their IRB to collaborate with PIs as much as possible. “We encourage the reviewers to work constructively with the reviewees, not have one of those hands-off things.” **IRB33**

Chairs may push for open doors because of their personal, political, and/or bureaucratic philosophy. Some chairs were wary of bureaucracies. (“We are easy, and try to be accessible.” IRB5) Another chair, who is a lawyer, tries to avoid having to “police” researchers. “I really hate having to deal with compliance issues. I hate being the enforcer…That’s not fun. That’s not what I do this for.” **(IRB19)**

Other factors, such as structural space parameters, can also play important roles in shaping IRB-PI relationships. As one IRB administrator said,

"At times, it has more to do with physical office design: we don’t have a receptionist. When we had someone between us and the world, PIs said that I “suddenly started screening calls,” or “didn’t have an open door policy anymore.” Now, researchers feel they can just come in and sit down, and we can talk about their protocol – what they haven’t addressed, whether they can address it, and whether I can explain to the board why it’s not there – that it’s on its way. **IRB13**"

At many institutions, interviewees complained that their IRBs invited PIs to meetings, but that these PIs often did not attend. These interviewees felt that such attendance could improve relationships – showing that IRBs are trying to be reasonable. Chairs may be surprised that researchers do not accept these invitations.

"It’s an open forum meeting. We tell all researchers to come. But they won’t. It’s a two or three-hour evening meeting, though they don’t have to come to the whole thing. It would help if they could look around the table and see their colleagues, and people from the lay community, and clergymen: *this isn’t a group of cynics and big red pens*. We really discuss the issues. Researchers just need to realize that this is what actually happens during the meetings, and we’re all pretty reasonable. **IRB27**"

Some chairs thus try to have PIs not see IRBs simply as faceless bureaucrats.

Such joint meetings can also prompt IRBs to be more sensitive to researchers.

"It’s tempting, and easier, to sit around a table and be really tough and critical and blunt when we have some anonymous individual – we’ve got a name there, but nobody knows them. When you have met the individual, you tend to temper those kinds of remarks. Even if ultimately the message is the same, we’ve backed off on the brute force. **IRB21**"

It may be harder for IRBs to be overly harsh and callous to a PI when meeting him or her in person, as opposed to interacting only through memos.

Yet other IRBs may prefer to close their meetings, or struggle to determine what to do. One administrator said, “In the past, the IRB would have investigators go to the meeting. Now they’ve closed them, which is helpful.” **(IRB23)**

Several IRBs established other institutional structures as well, such as “clinics,” to address PI concerns outside of formal reviews, *per se*.

"We started doing clinics. Researchers can meet with a subset of the IRB, and talk about what they want to do, and how to write their protocol. That helped. It was in a sense a *pre-review*, but also worked through questions that stymied the investigator from putting the protocol in. Mostly, [researchers] were afraid it wouldn’t get through. Everybody really appreciated it – both the IRB members (because they got better protocols), and the investigators (because they better understood the processes, and could then write better protocols). **IRB28**"

Such meetings and workshops may enhance openness, transparency, and communication.

"The clinics showed the investigators what we’re thinking, and brought them into the process, rather than just dropping the protocol off in some box, with a closed door. **IRB28**"

To improve relationships, other IRBs established on-line or in-print newsletters with updates – e.g., a “tip of the week.” As one chair said,

"We did a newsletter to keep people up-to-date, because they are not in the *IRB world*, and don’t know something has changed until it gets sprung on them. They said, “We’d like to know ahead of time what’s going on.” So, we established “The Tip of the Week.” It was easy to email: write it once, click a button, and send. **IRB4**"

IRBs can also disseminate such “tips” in response to errors that PIs may make. Thus, such advice can also potentially help prevent problems. This chair continued,

"A couple of investigators changed a study, and the IRB didn’t become aware until the protocol came up for renewal. So, we sent out a “Tip of the Week” that said you can’t do that! “But if a subject is on site, and a procedure needs to be repeated, and the PI’s best medical judgment is that it needs to be repeated, then repeat it. Don’t wait for IRB approval.” We tried to put federal regulations into a real world environment. **IRB4**"

Such advice may thus be helpful in several ways.

Yet these efforts can consume much of a chair’s or administrator’s time. A chair who spends a lot of time with PIs to diminish the IRB’s reputation of being obstructionistic may find that these activities take more time than he or she is compensated for. The chair above said his time is billed at 20%, but is really 35–40%.

"I spend more time with investigators than do any of our members, because I am so sick and tired of hearing that the IRB is a roadblock or a stumbling block. I don’t like the committee having that reputation. So I work very hard with our investigators to make that not the case. **IRB4**"

The time demands of such enhanced IRB availability can necessitate difficult tradeoffs, and cause tensions. IRB chairs and administrators can become overwhelmed, and need to weigh the advantages of open doors *vs.* limited resources.

Hence, while some chairs are highly concerned about PI complaints and try hard to reduce these, other chairs may be far less responsive or flexible. The latter may remain more removed from PIs, and interact with them more indirectly. IRBs may also struggle to achieve a balance. Boards may try to adopt an overall “open” policy, but adjust and limit their approach with difficult PIs over time.

"Some people are chronically unhappy – complainers. No matter what we do, they’re unhappy. So, we try to be diplomatic, gracious, and non-confrontational, but hold our ground. We’re not going to cave in just because somebody is yelling at us. We can’t turn the world on its head because a PI got his protocol in late! **IRB40**"

### Changing the tone and content of interactions

#### Helpful memos: the content of communications

IRBs often sought to improve, too, the quantity, quality, and helpfulness of written communication with individual PIs, but varied in how and to what degree they did so. Both the content and the tone of communication and interactions can be important. Some chairs write lengthy memos to PIs in response to submitted protocols, assisting these PIs in rewriting studies. Yet these activities can take time, and chairs and staff may therefore carefully choose to which PIs and studies to devote such efforts. As one administrator said, “Some people just want you to write the darn thing for them. That’s not my job.” **(IRB23)**

Hence, if a PI has a track history of poorly written proposals, the IRB may invest less such effort.

"For a very flawed study, I am much more likely to write back a three-page letter with 20 points, basically re-writing the protocol for them, than I am to disapprove it. I disapproved one from an investigator with a track record of submitting flawed protocols, and sloppy work – making us do a tremendous amount of work. Instead of doing the work for him for the fifth time, I said, “No. Here are the major problems. Fix it.” Instead of a three-page letter, I write a half-page letter, identifying the major issues, and put the ball back into their court. **IRB40**"

However, for various reasons, PIs may not all respond to these efforts as IRBs expect or hope. Interviewees felt that PIs revise and resubmit most, but not all, protocols. After receiving the three-page memo mentioned above, this chair said that the PI never resubmitted the proposal.

"I wrote an extensive, helpful letter, and he never responded. My guess is that he couldn’t fix it, and was overwhelmed. He is not a good researcher. But, for 99% of researchers, the concerns are fixable. They fix them, respond, and move on. **IRB40**"

#### Charm: establishing the right tone in communications

IRBs also attempt to mitigate friction with individual PIs through not only the *content* of communications, but the *tone* as well. Several chairs tried hard to use a respectful tenor that gave the message that the IRB wishes to be helpful, not obstructionistic; but such an approach was not always easy to establish and maintain.

Several interviewees described the importance of having what one chair calls a “deft touch.”

"I always fear that faculty feel worn down by regulations, and don’t have enough time for anything. I just try to get them to keep true to the IRB’s mission, and not just dismiss it as a whole set of hoops they have to jump through. Sometimes that just requires *a deft touch*. I don’t know if I’m even good at it – I hope I am. But that’s what I try to do. I’d much rather somebody get a phone call from me, since I’m also a doctor, and have done research, than just hear from somebody in the office that they didn’t do something. **IRB32**"

Chairs may thus also be uncertain as to how effective they are in these efforts. Achieving this tone, while simultaneously ensuring that PIs are following the regulations, can also be among the most difficult aspects of IRB work – doing PR while trying to protect subjects as much as possible.

"The hardest part of being an IRB administrator is walking this fine line. We are facilitators as well as monitors, and maintain a positive PR – we’re here to facilitate and help you with the process. At the same time, we’re here to make sure you comply with the regulations. Keeping the line of communication and trust open is critical, and setting the *tone*. **IRB16**"

IRB chairs may thus struggle to have their staff use “the right manner” to improve relationships with PIs, but establishing and maintaining such a tone may in part be an innate ability that not everyone equally shares.

"It’s a challenge not only to find folks who have the talent for IRB work, but to teach people to get the right tone and balance, not shaking a stick at researchers, but trying to be collegial, and knowing when and how to make exceptions, be flexible, or compromise. *We don’t want to be one of those IRB offices that people hate*, and complain about all the time. **IRB18**"

To strengthen relationships with PIs, and defuse friction, one chair tries to “say no with a smile” (IRB29) for unrealistic requests for rushed approval. An administrator, originally from the South, uses “Southern charm” and a sense of humor. When PIs don’t turn in paperwork, she says she tries to take the blame, rather than confronting them with their negligence.

"PIs will say they brought paperwork over, and I know they didn’t. They are sure. So most of the time, if there was an error, or they didn’t send something, I try to be first to say, “You brought that consent form to me the other day, and I have absolutely no idea what I’ve done with it. Could you send me another one, please?” It doesn’t really bother me anymore. PIs will backdate memos to the IRB, but we have a time-clock. I’ll say: “I know you intended to get that over here. I’m so sorry. Can we deal with it now? How can we help you *today*?” I say that because I’m not saying that they didn’t turn it in. *I’m trying to give them an out* so that they don’t have to say, “I promised that, but have no idea what happened!” It works a lot better for me to say, “I’m so sorry: it was in my hands and I must have misplaced it. Do you have another copy?” rather than, “Oh, we never received it.” They know they didn’t bring it. But we say, “We have looked high and low for that, but if you bring us another copy this afternoon, we’ll see if we can work it into the schedule.” It just doesn’t do any good to make demands. Let’s just move forward and see what we need to do to get everything running again. **IRB13**"

Thus, IRBs can seek to defuse potential tensions before these erupt.

#### Educating PIs

Interviewees felt that PIs and research staff range widely in quality and quantity of prior education concerning research ethics and IRB procedures. Interviewees thought that institutions vary in whether they require training in the Responsible Conduct of Research (RCR) for all staff involved in all research, and if so, how much. Several interviewees want the federal government to mandate comprehensive training more clearly, “requiring education in human subjects protection for everybody” **(IRB26)**.

Since IRB members sensed deficits in investigators’ training in these areas, “research ethics training,” now formally required by many institutions, may often consist merely of relatively short on-line exercises. Key aspects of issues, such as definitions of “adverse events,” may not be included in Good Clinical Practices or Responsible Conduct of Research courses. Yet interviewees felt that researchers may resent additional requirements.

Research conducted by *trainees* may pose particular challenges. Interviewees were often unsure where and to what degree junior PIs have learned about research ethics. Interviewees thought that at some institutions, residents and other trainees were mandated to do research, but may lack adequate methodological or research ethics training. IRBs often felt that some trainees may have deficient education in appropriate research design (e.g., as to whether the sample size is appropriate to warrant the study). “Residents say, ‘My faculty mentor told me what I need for the IRB,’ and the mentor is somebody I’ve never heard of, who’s never done research.” (IRB13)

#### Showing PIs the regulations

Some IRBs try to explicitly show PIs the regulations, to demonstrate that the IRB is not arbitrary in its use of power.

"When you can tell a researcher, “The FDA says, ‘No, you can’t do this,’ or ‘You should do this,’ or ‘have to do this,’” they understand. If you can show them the regs, they’re even happier. **IRB25**"

Other chairs explain to PIs not just the regulations, but the larger underlying ethical principles as well.

"There’s nothing worse than saying, “The regulation’s required.” You always have to tie it back to an ethical principle – say not only *what* they need to do, but *why*. It’s not *what* you say, it’s the *way* you say it. **IRB26**"

Again, how IRBs communicate can thus play important roles, but can vary. Not all IRB staff may offer such broader explanations.

Interviewees also felt that challenges existed in getting PIs to appreciate the ethics underlying the regulations. At times, interviewees thought that researchers resisted, or were not interested in these explanations. The guidelines themselves could also shift over time.

"Getting faculty or investigator buy-in, so that they understand *the reason* behind the regulation, is a challenge – being current on regulations and institutional and regulatory *expectations*. **IRB28**"

IRBs often felt that many PIs simply completed the necessary paperwork as requirements, not thinking of these documents as concerning larger ethical principles.

"They don’t think through the reasons why there’s compliance, or put it in an ethical context. Whether the answers are right or not may not matter to them. **IRB28**"

Several interviewees felt that IRB procedures and protections can themselves evolve to defend the institution from liability more than protect the well-being of subjects – thereby undermining PI dedication to these processes as ethically important.

"Optimally, there would be an ethics and integrity arm of research, and the compliance committees’ forms under that – bathed in *ethics*, rather than in institutional protection. **IRB28**"

Encouraging PIs to appreciate the principles underlying IRB concerns or requests regarding a particular study may also require resources that IRBs may lack. This former chair continued,

"Everything would come out better if a staff person would spend time with each investigator to improve understanding of the ethics behind the regulation, and help them write protocols in a more informed way. The science would be better, the subjects would end up better, and the investigator would feel a lot more buy-in. **IRB28**"

## Conclusions

This study, the first to explore the mechanisms through which IRBs respond to tensions with PIs, suggests that these committees react to conflicts with PIs in a variety of ways – both formal and informal, involving both the form and content of interactions and communications. These boards differ in how and to what degree they explicitly address conflicts with PIs – from proactively helping researchers respond to ethical concerns, to being less involved, and more anonymous. These data suggest that IRBs can potentially improve relationships with PIs in several ways, using more “open doors” rather than anonymity, engaging in outreach (e.g., through clinics), enhancing the tone as well as content of interactions, educating PIs about the underlying ethics, and helping PIs as much and proactively as possible. Still, these efforts can require resources, and encounter PI resistance.

While prior studies of IRBs have tended to be quantitative, and to view IRBs as static, the present data illuminate how these committees interact with PIs within the context of dynamic and evolving relationships with individuals PIs. These data thus highlight how IRBs operate as part of complex social systems, and serve as critical mediators between federal regulations and individual researchers.

IRBs face tensions partly because PIs may in effect “blame the messenger” (the IRB) for the news (that these regulations need to be followed). As IRBs have certain power [21], they can thus encounter challenges in establishing the right balance in these relationships. Since IRBs monitor PIs, these committees’ PR efforts may be suspect. Undoubtedly, PIs are also very busy, and confront many competing demands for their time, making it difficult for them to attend meetings to which IRBs invite them. Moreover, while many chairs would like PIs educated to “understand the ethics behind the regulations,” researchers may disagree with an IRB’s interpretation and application of these regulations. While a common adage is that “good ethics makes good medical care,” some interviewees extend this notion to research as well, feeling that good research ethics also make good science. Yet whether this aphorism pertains to all interpretations and applications of research ethics is unclear

As bureaucracies, IRBs vary not only in the formal social structures they establish, but in their tone and attitudes, which can shape how communication occurs, both formally and informally. IRBs face choices concerning how, when, and to what degree to interact and communicate with PIs, monitoring and seeking to shape these researchers’ attitudes and behaviours. A lack of transparency (*vs.* openness) can exacerbate PI frustrations with, and demonization of, IRBs. It may be easier to demonize an “anonymous” bureaucracy, rather than fellow human beings. IRBs, more than researchers, may prefer the lack of transparency and argue that keeping minutes private is easier than making this documents more available and redacting details. But, that objection may not sufficiently offset the potential benefits of openness in fostering ethical behaviour. Redaction may be possible. These interviewees thus raise questions of how much anonymity is or should be permitted.

This study suggests needs for increased attention to not only *what* IRBs communicate to PIs, but *how* (i.e., the tone and the nature of interactions), and to examine the lived experiences of IRBs and PIs – the ways interactions about ethics are carried out that can thus shape the effectiveness of these interactions.

Federal regulations do not explicitly discuss these issues, and some IRBs have developed their own approaches that can potentially be adopted more widely. In part, tensions may exist between IRBs and PIs because of underlying conflicting priorities – pursuing research *vs.* protecting subjects. The strategies presented here (e.g., “open doors”) may not wholly eliminate tensions, but can help.

For several reasons, including desires to improve relationships between IRBs and PIs, the ANPRM seeks to make formal structural changes (e.g., increasing the use of central IRBs in multi-site studies) [6,9,22]. But the present data suggest that not only the formal *structure*, but the *content* and *tone* of interactions are crucial. For full board reviews of multisite studies, for instance, centralization alone thus may not resolve all tensions. To reduce these strains, these data suggest that informal behaviours and attitudes of both IRBs and PIs should also shift.

While limited resources may restrict these committees in certain ways, other IRB approaches to enhancing relationships with PIs require relatively little time and energy (e.g., adopting an effective tone, and sending newsletters or “tips.”). Hence, the *status quo* can be improved by encouraging IRBs to try to ameliorate strains with PIs by adopting such approaches. Specifically, IRBs should realize more fully that they have a certain degree of latitude in their interactions with PIs, and can strengthen these relationships through not only what, but *how* they communicate. If an IRB lacks resources to institute some of these practices, the chair can potentially present these possible approaches to institutional leaders, highlighting the benefits, in hopes of garnering additional funds.

These data thus underscore the importance of trying to enhance IRBs and their interactions with PIs through not only formal macro policy (e.g., altering federal regulations, and establishing centralized IRBs), but more *informal* micro levels as well – at the level of daily interactions and lived experiences.

As a potential solution to many of these problems, the Institute of Medicine (IOM) report of almost 10 years ago supported IRB accreditation, which has since spread widely. Yet the current data suggest limitations in accreditation: it provides standards for formal mechanisms, but does not address many aspects of how IRB personnel in fact *fill* these functions. The present data suggest that implicit and explicit attitudes and tone need to be addressed as well, perhaps through further, finer-grained, and more nuanced education, and heightened awareness of these issues. Moreover, IRBs face challenges in part because the system is not static, but fluid, with new scientific methods and new trainees.

Future research is needed to examine more fully how IRBs work within these dynamic relationships with researchers – how often, when, in what ways, and to what degrees IRBs in fact adopt the range of approaches described here; how successful each of these strategies are, and can be; and what factors are associated with IRB decisions to adopt or avoid these techniques. In part, IRB chairs may make these choices based on the degrees to which they and/or their members are “pro-research;” and their prior attitudes and perceptions concerning relationships with PIs. Future studies can thus probe more fully how IRBs make these decisions, and whether and how the approaches presented here can lower tensions, and thereby enhance PI cooperation and compliance with regulations, improving human subject protection.

The potential benefits of creating cultures of “compliance” and of “conscience” have been described [23-25], but whether, when, how, and to what degree IRBs actually do or can achieve such goals has received little, if any attention. The present data suggest that IRBs face a range of options for facilitating such aims, but do not always adopt or follow these methods.

These data also have important implications for professional education of IRB chairs, members and staff, research investigators and staff, and trainees – to enhance their interactions as effectively as possible.

This study has several potential limitations. These interviews explored subjects’ views now and in the past, but not prospectively over time, to explore changes. Participants’ statements reflect their views and attitudes, and do not necessarily represent objective “fact” *per se*, but are nonetheless valuable in and of themselves. Interviews also did not include PIs at each of the institutions contacted. However, future studies can employ these approaches. These data reflect on in-depth interviews with IRB chairs and members, and did not include direct observation of IRBs engaged in meetings, or of written IRB records. Future research can observe IRBs and examine such documents. Yet these added data may be hard to procure since, anecdotally, IRBs have often required researchers to obtain consent from all IRB members, the PIs, and protocol funders.

In sum, IRBs range considerably in whether, to what degree, and when they adopt strategies that may potentially reduce tensions with PIs. Federal regulations do not mention these approaches; yet increased awareness of the range and benefits of these strategies can potentially improve IRB interactions with researchers, thus enhancing the dual goals of promoting socially beneficial science, while protecting study participants.

## Appendix A

### A. 1. Sample questions from semi-structured interview

Note: Additional follow-up questions were asked, as appropriate, with each participant.

▪ How do you define research integrity (RI)? Do you think IRBs and PIs view or approach RI differently, and if so, how, when, and why? Have you seen problems in PI non-compliance with IRB regulations? If so, what kinds of problems? How do you address these?

▪ What kinds of tensions, if any, has your IRB faced with PIs concerning RI or other, related issues?

▪ Has your IRB ever tried to address these tensions? If so, how, when, why, and with what success? What barriers, if any, did you encounter? What, if anything, has helped in addressing these tensions with PIs? How, when, why, and with what result?

▪ Do you have any other thoughts about these issues?

## Competing interests

The authors declare that they have no competing interests.

## Authors’ contributions

RK designed and conducted the study, and wrote and approved the final manuscript.
